# Ascorbic acid, corticosteroids, and thiamine in sepsis: a review of the biologic rationale and the present state of clinical evaluation

**DOI:** 10.1186/s13054-018-2217-4

**Published:** 2018-10-29

**Authors:** Ari Moskowitz, Lars W. Andersen, David T. Huang, Katherine M. Berg, Anne V. Grossestreuer, Paul E. Marik, Robert L. Sherwin, Peter C. Hou, Lance B. Becker, Michael N. Cocchi, Pratik Doshi, Jonathan Gong, Ayan Sen, Michael W. Donnino

**Affiliations:** 1 Beth Israel Deaconess Medical Center, Department of Medicine, Division of Pulmonary, Critical Care, and Sleep Medicine, Boston, MA USA; 2 Beth Israel Deaconess Medical Center, Department of Emergency Medicine, Boston, MA USA; 3 Research Center for Emergency Medicine, Aarhus University Hospital, Aarhus, Denmark; 4 Department of Anesthesiology, Aarhus University Hospital, Aarhus, Denmark; 5 Department of Critical Care Medicine, University of Pittsburgh, Pittsburgh, PA USA; 6 Department of Emergency Medicine, University of Pittsburgh, Pittsburgh, PA USA; 7 Department of Internal Medicine, Eastern Virginia Medical School, Norfolk, VA USA; 8 Department of Emergency Medicine, Wayne State University School of Medicine/Detroit Receiving Hospital, Detroit, MI USA; 9 Division of Emergency Critical Care Medicine, Department of Emergency Medicine, Brigham and Women’s Hospital, Boston, MA USA; 10 Department of Emergency Medicine, Donald and Barbara Zucker School of Medicine at Hofstra/Northwell, Hempstead, NY USA; 11 Feinstein Institute for Medical Research, Manhasset, NY USA; 12 Department of Anesthesia Critical Care, Division of Critical Care, Beth Israel Deaconess Medical Center, Boston, MA USA; 13 Department of Emergency Medicine and Internal Medicine, University of Texas Health Science Center at Houston, Houston, TX USA; 14 Donald and Barbara Zucker School of Medicine at Hofstra/Northwell, Northwell Health System, New Hyde Park, NY USA; 15 Department of Critical Care Medicine, Mayo Clinic, Phoenix, AZ USA; 16 Beth Israel Deaconess Medical Center, Emergency Medicine, One Deaconess Rd, W/CC 2, Boston, MA 02215 USA

**Keywords:** Thiamine, Ascorbic acid, Corticosteroids, Metabolic resuscitation, Sepsis

## Abstract

The combination of thiamine, ascorbic acid, and hydrocortisone has recently emerged as a potential adjunctive therapy to antibiotics, infectious source control, and supportive care for patients with sepsis and septic shock. In the present manuscript, we provide a comprehensive review of the pathophysiologic basis and supporting research for each element of the thiamine, ascorbic acid, and hydrocortisone drug combination in sepsis. In addition, we describe potential areas of synergy between these therapies and discuss the strengths/weaknesses of the two studies to date which have evaluated the drug combination in patients with severe infection. Finally, we describe the current state of current clinical practice as it relates to the thiamine, ascorbic acid, and hydrocortisone combination and present an overview of the randomized, placebo-controlled, multi-center Ascorbic acid, Corticosteroids, and Thiamine in Sepsis (ACTS) trial and other planned/ongoing randomized clinical trials.

## Background

Sepsis is a common and highly morbid condition with an estimated 1.7 million cases occurring in the United States each year, resulting in over 270,000 deaths [1]. Despite advances in critical care practices, sepsis remains the most common cause of death in non-cardiac intensive care units (ICUs) [2, 3]. Even among sepsis patients who survive their hospital stay, residual organ dysfunction requiring ongoing treatment after discharge is common [4]. Despite this high level of mortality and morbidity, antibiotics and source control remain the only focused therapies for this condition [5]. In a small, retrospective observational study of septic ICU patients, the combination of thiamine (200 mg every 12 h), ascorbic acid (1500 mg every 6 h), and hydrocortisone (50 mg every 6 h) was associated with a dramatic improvement in organ injury, time to shock reversal, and mortality as compared to historical controls at the same hospital [6]. Each component of this combination of therapies has been recently evaluated individually in septic shock patients. A prior pilot randomized trial found that the provision of thiamine to septic shock patients with elevated lactate attenuated organ dysfunction (particularly renal dysfunction) and reduced lactate levels and potentially mortality in those patients with baseline thiamine deficiency [7, 8]. In addition, two small randomized trials of ascorbic acid vs placebo in sepsis have demonstrated improved clinical outcomes [9, 10]. Finally, while there have been mixed results with respect to the benefit of corticosteroids in septic shock generally [11, 12], the addition of corticosteroids to ascorbic acid may have a synergistic effect [6, 13–15].

In the present article, we review the biologic basis for and existing data supporting the use of thiamine, ascorbic acid, and corticosteroids in sepsis. We discuss the use of this drug combination in current clinical practice and the rationale for the currently enrolling Ascorbic Acid, Corticosteroids, and Thiamine in Sepsis (ACTS) trial, as well as other clinical trials addressing this question.

## Organ dysfunction in sepsis

The traditional paradigm of organ dysfunction in sepsis has focused on decreased systemic vascular resistance resulting in decreased organ perfusion, and ultimately impaired oxygen delivery [16–18]. Numerous studies, however, have shown that organ dysfunction can occur during sepsis and septic shock even in the absence of decreased perfusion [19–21]. Notably, histopathologic analyses of organs following death from sepsis often fail to demonstrate any substantial amount of ischemic injury, but rather reveal remarkably preserved parenchyma or a predominant pattern of apoptosis, suggesting alternative mechanisms of organ dysfunction apart from hypoperfusion and independent of cellular oxygen delivery [21–23]. A number of such mechanisms have been proposed and include mitochondrial dysfunction with resultant bioenergetic failure, a direct effect of the immune response to infection (related to pathogen-associated and damage-associated molecular patterns), microvascular abnormalities, endothelial dysfunction, and inter-organ cross-talk [24, 25].

## Thiamine, ascorbic acid, and corticosteroids

**Fig. 1:**
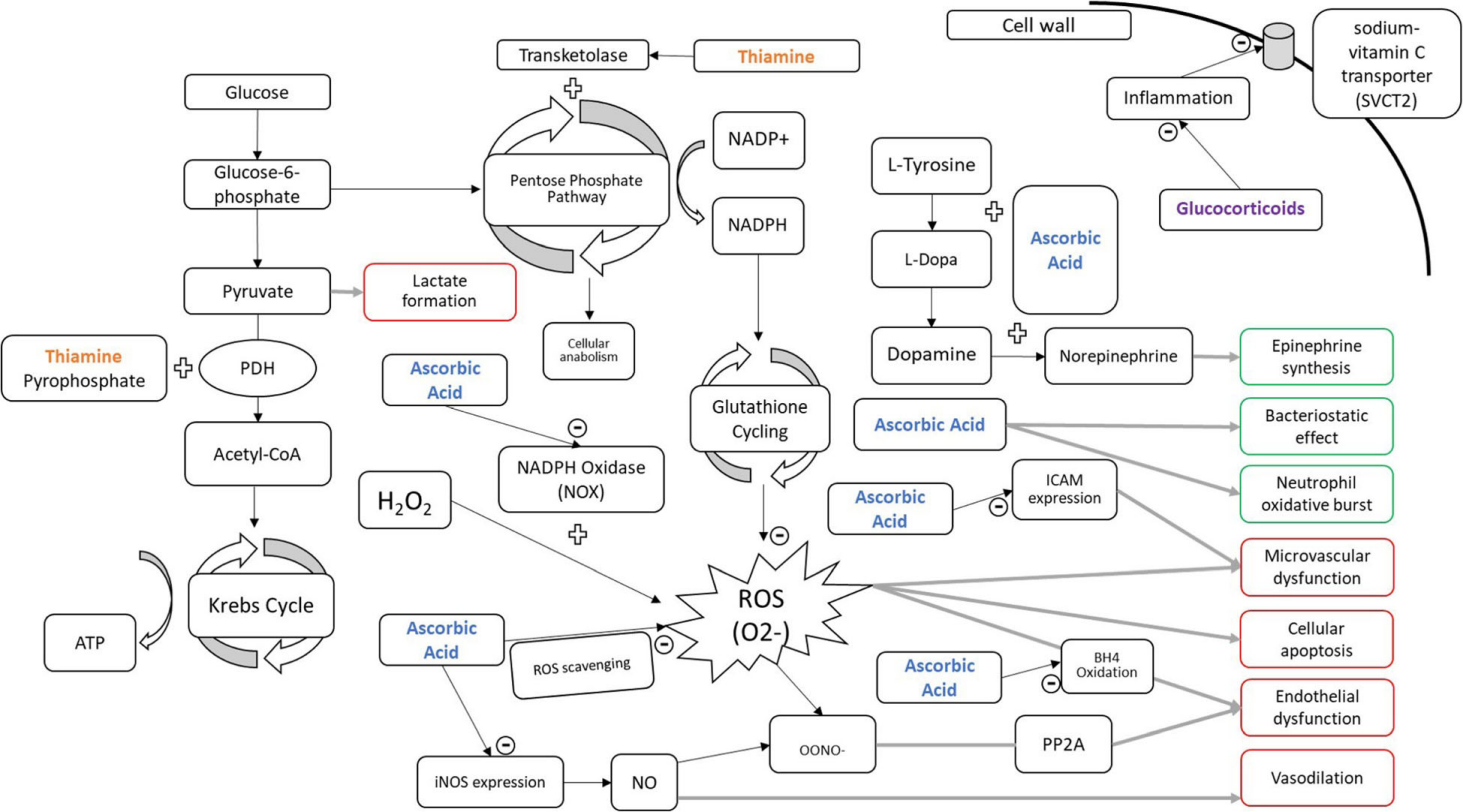
Suggested mechanisms for the efficacy of thiamine, ascorbic acid, and corticosteroids in sepsis. *PDH* pyruvate dehydrogenase, *ATP* adenosine triphosphate, *NADPH* nicotinamide adenine dinucleotide phosphate, *PP2A* protein phosphatase 2, *ROS* reactive oxygen species, *BH4* tetrahydrobiopterin, *ICAM* intracellular adhesion molecule. A *circled minus sign* indicates an inhibitory action; *arrows* indicate an activating action; *green-outlined boxes* indicate a beneficial effect of the medication combination; *red-outlined boxes* indicate a potentially harmful effect attenuated by the medication combination

The current management of sepsis and septic shock largely focuses on improving oxygen delivery via a combination of intravenous fluid and vasoactive medications while treating the infection with antibiotics and source control [5]. The combination of thiamine, ascorbic acid, and corticosteroids has been suggested as a potential adjunctive therapy targeted at non-oxygen delivery-dependent mechanisms of organ dysfunction (see Fig. 1 for a summary of suggested mechanisms).

### Thiamine

Thiamine (vitamin B1) is a water-soluble vitamin that is a key component of a number of cellular metabolic processes. In its phosphorylated form, thiamine pyrophosphate, thiamine acts as a cofactor for pyruvate dehydrogenase, the enzyme necessary for converting pyruvate to acetyl-coenzyme A for entry into the Krebs cycle. When thiamine levels are insufficient, pyruvate is unable to be converted to acetyl coenzyme A, resulting in impaired aerobic respiration and a compulsory shift to the anaerobic pathway, resulting in elevated serum lactate levels [26–28]. Thiamine also plays a role in the metabolism of branched-chain amino acids and is a critical component of the pentose phosphate pathway, which is essential for the generation of NADPH and therefore glutathione cycling, an important anti-oxidant pathway [29–31]. A thiamine deficiency syndrome, beriberi, bears a number of similarities to sepsis, including peripheral vasodilation, cardiac dysfunction, and elevated lactate levels [29].

Thiamine deficiency is not rare in critically ill populations and may be associated with increased mortality in some cases [7, 29, 32, 33]. Further, thiamine levels are depleted during the course of critical illness and the administration of thiamine during critical illness may improve organ dysfunction [34–36]. In a mouse model of cardiac arrest, the provision of thiamine improved mitochondrial function, reduced histologic signs of brain injury, and improved neurologic outcomes [37]. In a canine model of septic shock, thiamine pyrophosphate improved lactate clearance, oxygen consumption, and arterial pressure irrespective of thiamine deficiency status [38]. In the only randomized trial of thiamine in human septic shock, 88 patients were selected for increased risk of symptomatic thiamine deficiency based on a serum lactate > 3 mmol/L after volume resuscitation. In that study, there was no effect overall of thiamine on the primary outcome of median lactate level at 24 h, though there was a statistically significant difference when evaluating lactate levels at serial timepoints in the first 72 h. In a pre-defined subgroup of patients with thiamine deficiency (35% of the cohort), however, the administration of thiamine reduced lactate levels and improved mortality [7]. In a post hoc analysis of that study, patients without baseline end-stage renal disease who were given thiamine had better renal outcomes than those randomized to placebo [8]. Thiamine supplementation has not been shown to be associated with significant adverse effects, even at high doses [39]. Overall, these results suggest a role for thiamine supplementation as a low-risk and potentially high reward intervention for some patients with septic shock and increased baseline risk of thiamine deficiency.

### Ascorbic acid

Like thiamine, ascorbic acid is a water-soluble vitamin essential for a number of processes in the human body. As an anti-oxidant, ascorbic acid is an electron donor that directly scavenges free radicals, prevents the generation of new free radicals through its suppressive effects on the NADPH oxidase (NOX) pathway, and assists in the recycling of other anti-oxidants [40–42]. The anti-oxidant effect of ascorbic acid results in reduced endothelial permeability, improved microvascular and macrovascular function, and attenuated cellular apoptosis in pathological states [41, 43, 44]. In addition, ascorbic acid has a number of effects on the immune system, including regulation of macrophage function, reduction of inflammatory mediators, and even a direct bacteriostatic effect at high concentrations [45–47]. Lastly, ascorbic acid is essential in the generation of endogenous vasopressors and may be important in maintaining vascular vasopressor responsiveness [41, 48].

Prior studies have found that plasma and cellular levels of ascorbic acid decline rapidly during critical illness [49–51]. Similar to thiamine, ascorbic acid deficiency syndrome (scurvy) bears a number of similarities to sepsis, including malaise, coagulation abnormalities, and endothelial wall breakdown [49]. While interest in ascorbic acid for the management of critical illness has recently been reinvigorated, it is not new. The potential benefit of ascorbic acid for reducing resuscitation fluid requirements in burn patients and organ dysfunction in critically ill surgical patients was suggested over a decade ago [52, 53]. More recently, small randomized trials in sepsis have shown promise. In one study, 24 septic patients were randomized in a 1:1:1 ratio to receive high dose ascorbic acid (200 mg/kg), low dose ascorbic acid (50 mg/kg), or placebo. In that study, no adverse effects were related to the ascorbic acid and patients who received ascorbic acid had more rapid reduction in measures of organ injury, inflammation, and procalcitonin. There also appeared to be a dose–response relationship, with patients who received higher dose ascorbic acid having more rapid clinical improvement [9]. In a more recent study, 28 patients with vasopressor-dependent septic shock were randomized to 25 mg/kg of ascorbic acid every 6 h or placebo [10]. Those in the ascorbic acid arm required lower vasopressor doses and had lower mortality.

While the high doses of ascorbic acid given in the above clinical studies were not associated with any identified harms specific to the drug, one theoretical concern regarding the routine use of ascorbic acid in sepsis is the potential for increased oxalate excretion and the development of oxalate renal calculi [54]. Thiamine pyrophosphate is a key co-enzyme necessary for the function of glyoxylate aminotransferase, which catalyzes the breakdown of glyoxalate to carbon dioxide instead of oxalate. Thiamine deficiency states, therefore, may predispose to increased oxalate excretion [54, 55]. It should be further noted that short-term, intravenous ascorbic acid—even at high doses—has not been found to increase the risk of renal calculi in controlled trials to date [41, 43]. Other potential adverse effects of vitamin C include abdominal pain/bloating, increased iron absorption, hemolysis in patients with G6PD enzyme deficiency, and false negative results on fecal occult blood testing [56]. At very high doses, ascorbic acid may act as a pro-oxidant, although this has not been found to be the primary effect in vivo [41, 57]. Finally, high doses of ascorbic acid may falsely elevate glucose level readings when measured with certain point-of-care glucometers employing glucose dehydrogenase-pyrroloquinoline quinone amperometric methods [58].

### Corticosteroids and ascorbic acid

A number of large, randomized trials have assessed the added benefit of corticosteroids when included as part of general septic shock management. These studies have generally shown corticosteroids to improve various clinical outcomes in septic shock (e.g., time to shock reversal, ventilator-free days), but there have been mixed results with respect to mortality [59–62]. Whether the routine administration of hydrocortisone to patients with septic shock should be standard remains a matter of debate [11, 12].

The biologic basis for the inclusion of hydrocortisone in the drug combination, however, is based on potential synergy between ascorbic acid and hydrocortisone. Glucocorticoid binding to glucocorticoid receptors is negatively affected by oxidizing molecules. This may be reversed by the administration of ascorbic acid, which has been shown to restore glucocorticoid receptor function [13]. The cellular uptake of ascorbic acid is mediated by the sodium-vitamin C transporter (SVCT2), which is downregulated during inflammatory states. The administration of glucocorticoids has been shown to increase expression of the transporter [14, 15]. In a study examining the barrier function of human lung microvascular epithelial cells, the combination of ascorbic acid and hydrocortisone showed a synergistic barrier-protective effect after lipopolysaccharide exposure—above the combined effect of either agent when given alone [15].

### Thiamine, ascorbic acid, and corticosteroids

The combination of thiamine, ascorbic acid, and corticosteroids has been studied in two, single center, before-and-after cohort studies [6, 63]. In addition to the above-referenced study by Marik et al., a recent study performed in South Korea compared 53 patients with severe pneumonia admitted to the ICU who received the thiamine, ascorbic acid, and hydrocortisone combination to historical controls. In that study, patients who received the thiamine, ascorbic acid, and hydrocortisone combination had a substantial mortality benefit (adjusted odds ratio 0.15, 95% CI 0.04–0.56). Although there were baseline imbalances in the ‘control’ and ‘treatment’ groups wherein patients in the ‘treatment’ group were more likely to have been receiving vasopressor and renal replacement therapy, the mortality benefit persisted after propensity-adjustment and propensity-matching. While these studies were the first to explore the drug combination in severe infection, their observational methodology, inclusion of non-consecutive and non-concurrent ‘control’ arms, small sample sizes, and single center nature represent significant limitations and preclude broad conclusions regarding the efficacy of this drug combination in sepsis.

## Ascorbic acid, corticosteroids, and thiamine for the treatment of sepsis in current practice

As detailed above, scientific support for various elements of the thiamine, ascorbic acid, and hydrocortisone drug combination has existed for decades. Enthusiasm for this drug combination in sepsis has grown rapidly since 2016 due to the aforementioned paper by Marik et al. and the significant exposure it has received in both specialty medical blogs and the lay press [64–68]. Reactions in specialty medical blogs were mixed, with some physicians supporting the incorporation of the ascorbic acid, corticosteroids, and thiamine drug combination into routine sepsis management [66] and others arguing for more rigorous testing of the drug combination [67, 68]. Arguments for more immediate uptake include the perceived low risk and relatively low cost of the intervention, biologic plausibility, and support from present literature, within the limitations that they represent. Arguments against include a long history of promising sepsis interventions that failed more rigorous scientific testing (e.g., activated protein C [69]), the unknown safety profile of high-dose ascorbic acid in critically ill populations (and in combination with corticosteroids and thiamine), and a general concern regarding the generalizability of results from single center observational studies. At present, our discussions with critical care leaders at a number of academic and community centers have found that practice patterns are mixed, with some clinicians opting for routine administration of the drug combination, others only administering the drug combination in sepsis patients who are decompensating despite traditional management, and others who do not administer the drug combination at all.

## The Ascorbic Acid, Corticosteroids, and Thiamine in Sepsis (ACTS) trial and other ongoing clinical trials

The lack of adequate data exemplified by mixed practice patterns suggests a state of scientific equipoise has developed regarding the risk/benefit ratio of the routine administration of ascorbic acid, corticosteroids, and thiamine in sepsis. Randomized clinical trials are urgently needed to assess the effect of this drug combination on clinically important outcomes in sepsis. As of July 1st, 2018, a review of the World Health Organization International Clinical Trials Registry Program (which includes clinicaltrials.gov) revealed nine ongoing or planned clinical trials of ascorbic acid, corticosteroids, and thiamine in six different countries (Table 1). These trials differ somewhat with respect to study populations (septic shock only vs sepsis or septic shock), control group interventions (the VITAMINS trial using hydrocortisone and the remainder choosing saline placebo), and primary outcomes, but all explore the intervention and daily doses proposed in the study by Marik et al [6]. Of note, the Vitamin C Infusion for Treatment in Sepsis Induced Acute Lung Injury (Citrus-ALI), a 170-patient trial of 200 mg/kg/day of ascorbic acid vs placebo in sepsis-induced acute lung injury, has completed enrollment, although results of this study are not yet available.

**Table 1:** Ongoing and planned clinical trials of thiamine, ascorbic acid, and corticosteroids, in sepsis

Trial name	Trial identifier	Country	Population	Primary outcome
Ascorbic acid, Corticosteroids, and Thiamine in Sepsis (ACTS) Trial	NCT03389555	USA	Septic shock	Change in SOFA score
Vitamin C, Thiamine and Steroids in Sepsis (VICTAS)	NCT03509350	USA	Sepsis with acute cardiovascular or respiratory compromise	Vasopressor- and ventilator-free days
Hydrocortisone, Vitamin C, and Thiamine for the Treatment of Sepsis and Septic Shock (HYVCTTSSS)	NCT03258684	China	Sepsis or septic shock (Sepsis-3 Criteria)	Hospital mortality
The Effect of Vitamin C, Thiamine and Hydrocortisone on Clinical Course and Outcome in Patients With Severe Sepsis and Septic Shock	NCT03335124	Slovenia	Severe sepsis or septic shock	Hospital mortality
Metabolic Resuscitation Using Ascorbic Acid, Thiamine, and Glucocorticoids in Sepsis (ORANGES)	NCT03422159	USA	Sepsis or septic shock	Hospital mortality
The Vitamin C, Hydrocortisone and Thiamine in Patients With Septic Shock Trial (VITAMINS)	NCT03333278	Australia and New Zealand	Septic shock	Vasopressor-free days
Evaluation of Hydrocortisone, Vitamin C and Thiamine for the Treatment of Septic Shock (HYVITS)	NCT03380507	Qatar	Septic shock	Hospital mortality
Steroids, Thiamine, and Vitamin C in Septic Shock (STACSS)	CTRI/2018/04/013384	India	Septic shock	Shock reversal
Thiamine, Vitamin C and Hydrocortisone in the Treatment of Septic Shock	NCT03540628	USA	Septic shock	Mortality (as compared to the study by Marik et al. [6])

Our study, the Ascorbic Acid, Corticosteroids, and Thiamine in Sepsis (ACTS) Trial is a multi-center randomized clinical trial in the United States aimed at assessing the effect of the drug combination on organ function and other outcomes in septic shock. The ACTS trial is coordinated by the Center for Resuscitation Science at Beth Israel Deaconess Medical Center (BIDMC) in Boston, MA, USA and is currently enrolling. The trial is supported by the Open Philanthropy Project (https://www.openphilanthropy.org/). Patients are randomized in a 1:1 ratio to receive thiamine (100 mg), ascorbic acid (1500 mg), and hydrocortisone (50 mg) or matching placebo four times daily for 4 days. The primary outcome is change in the Sequential Organ Failure Assessment (SOFA) score from baseline to 72 h, with key secondary outcomes including the incidence of renal failure and 30-day mortality. The primary outcome of 72-h SOFA score was selected to reflect the anticipated beneficial effects of thiamine, ascorbic acid, and corticosteroids on organ function. As organ dysfunction is a defining element of sepsis and a major determinant of survival, this outcome is patient centered and the attenuation of organ dysfunction may be practice changing. Further, the SOFA score can be measured early in a patient’s hospital course and is therefore less impacted by downstream elements of hospital care than overall mortality.

The ACTS trial, in combination with the other trials described above, may provide important validation of the results found by Marik et al. Should those results be replicated, the potential benefit in terms of lives saved world-wide annually from sepsis could be measured in the hundreds of thousands. Even if the results from Marik et al. are not replicated, the ACTS trial and other trials of thiamine, ascorbic acid, and corticosteroids will provide important scientific data regarding the effect of metabolic resuscitation in sepsis that may guide future studies in this area. Given concerns about reproducibility in science [70], replication of results in different patient populations is crucial to demonstrating a true, generalizable effect. The multiple ongoing trials testing this drug combination creates a rare scenario in critical care medicine research where multiple, independent investigators are exploring the effects of a single intervention in unique patient cohorts. Further, a prospectively planned patient-level metanalysis combining data from the ACTS trial and the VICTAS trial would provide increased power and an ability to better explore the effect of thiamine, ascorbic acid, and hydrocortisone in certain patient subgroups [71].

## Conclusions

The combination of thiamine, ascorbic acid, and corticosteroids is a promising new therapy for sepsis resuscitation but currently lacks robust evidence to support its widespread use. The potential effectiveness of this medication combination is rooted in biologic plausibility and supported by small clinical trials of the various individual components. Randomized data to confirm or refute the observational evidence for the drug combination are needed, and several clinical trials are ongoing or planned in the near future. We therefore anticipate a timely answer to the question of whether thiamine, ascorbic acid, and corticosteroids will play a role in the evolution of sepsis therapies.

## Abbreviations

ACTS: Ascorbic acid, Corticosteroids, and Thiamine in Sepsis
ICU: Intensive care unit
NOX: NADPH oxidase pathway
SOFA: Sequential organ failure assessment
